# *Leucosolenia qingdaoensis* sp. nov. (Porifera, Calcarea, Calcaronea, Leucosolenida, Leucosoleniidae), a new species from China

**DOI:** 10.3897/zookeys.906.47164

**Published:** 2020-01-22

**Authors:** Yan-Ling Chu, Lin Gong, Xin-Zheng Li

**Affiliations:** 1 Institute of Oceanology, Chinese Academy of Sciences, Qingdao 266071, China Institute of Oceanology, Chinese Academy of Sciences Qingdao China; 2 University of Chinese Academy of Sciences, Beijing 100049, China University of Chinese Academy of Sciences Beijing China; 3 Center for Ocean Mega-Science, Chinese Academy of Sciences, Qingdao, 266071, China Center for Ocean Mega-Science, Chinese Academy of Sciences Qingdao China; 4 Laboratory for Marine Biology and Biotechnology, Pilot National Laboratory for Marine Science and Technology (Qingdao), Qingdao 266237, China Laboratory for Marine Biology and Biotechnology Qingdao China

**Keywords:** Sponge, taxonomy, Yellow Sea

## Abstract

A new species of Leucosoleniidae, *Leucosolenia qingdaoensis***sp. nov.**, is described. This new species was collected in a scallop-breeding pond from the Yellow Sea and preserved in 75% ethanol. This sponge consists of a dense reticulation of ascon tubes, with the surface minutely hispid and the consistency soft and fragile.

Spiculation of the new species consists of diactines, which are smooth, straight or sometimes slightly curved, triactines of two types, and tetractines with short and curved apical actines; spiculation also slightly overlaps and is somewhat irregularly assembled. Together these form a thin layer of skeleton, with a small number of cells, which results in a transparent, white sponge. As a typical asconoid feature, all internal cavities of the sponge are lined with choanocytes, and there is no fully developed inhalant system. Comparisons with other *Leucosolenia* reported from the Pacific Ocean are also made.

## Introduction

The family Leucosoleniidae is characterised by a branched and rarely anastomosed cormus and asconoid aquiferous system; there is neither a common cortex nor a delimited inhalant or exhalant aquiferous system (Minchin 1900). The family includes three genera (Borojevic et al. 2002): *Ascyssa* Haeckel, 1872, *Ascute* Dendy & Row, 1913, and *Leucosolenia* Bowerbank, 1864. They can be easily distinguished by their skeletons: the skeleton of *Ascyssa* contains only diactines; the skeleton of *Ascute* exhibits giant longitudinal diactines forming a continuous layer on the external surface, and includes triactines and tetractines; and the skeleton of *Leucosolenia* lacks any of these obvious characteristics in the above two genera. Instead, the skeleton of *Leucosolenia* is characterised by being composed of diactines, triactines and/or tetractines, without a reinforced external layer on the tubes.

The genus *Leucosolenia* comprises 40 living species worldwide (Van Soest et al. 2019), of which only three species, *L. microspinata* Longo, 2009, *L. salpinx* Van Soest, 2017, and *L. parthenopea* Sarà, 1953, were named after 1950; 11 species were described by Haeckel between 1870 and 1872. The literature of this genus is relatively old, and the descriptions contained therein of the species of *Leucosolenia* were simple, almost without details and illustrations of the body shapes and spicules. Thus, a taxonomic revision of this genus is very difficult, and to date, no worldwide revision of the genus has been made.

**Figure 1.**
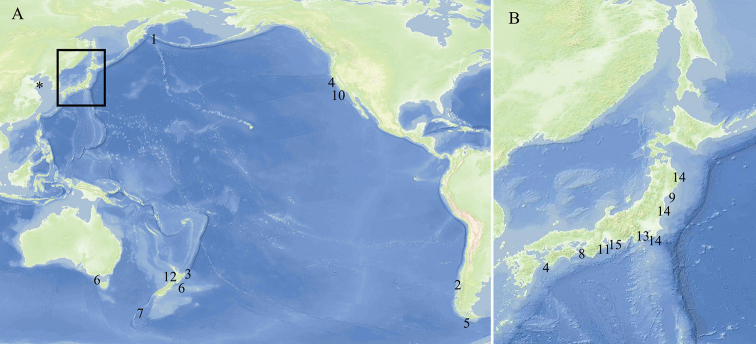
Distribution of *Leucosolenia***A** location in the Pacific Ocean **B** detail of the type locality in the Japanese coast: (1) Komandorski Islands (*L. albatrossi* Hôzawa, 1918); (2) Comau Fjord (*L. australis* Brøndsted, 1931); (3) Cook Strait, Poverty Bay, Kawakawa (*L. echinata* Kirk, 1893); (4) Francisco Bay, California; Sukumo ôsima, Kôti Prefecture, Sagimi Sea (*L. eleanor* Urban, 1906); (5) Tierra del Fuego (*L. feuerlandica* Tanita, 1942); (6) Port Phillip Heads, Australia, and New Zealand (*L. lucasi* Dendy, 1891); (7) Macquarie Island (*L. macquariensis* Dendy, 1918); (8) Wakayama Prefecture (*L. minuta* Tanita, 1943); (9) Onagawa Bay (*L. mollis* Tanita, 1941); (10) Monterey Bay, California (*L. nautilia* Laubenfels, 1930); (11) Mie Prefecture (*L. pyriformis* Tanita, 1943); (12) New Zealand (*L. rosea* Kirk, 1896); (13) Yodomi, Sagami Sea (*L. serica* Tanita, 1942); (14) Matsushima Bay, Onagawa Bay, Izushima (*L. tenera* Tanita, 1940); (15) Wagu Miye Prefecture (*L. ventosa* Hôzawa, 1940); (*) Qingdao (*L. qingdaoensis* sp. nov.).

The localities of the 15 known species of *Leucosolenia* recorded from the Pacific Ocean are shown in Figure 1. Seven species (*L. eleanor* Urban, 1906, *L. minuta* Tanita, 1943, *L. mollis* Tanita, 1941, *L. pyriformis* Tanita, 1943, *L. serica* Tanita, 1942, *L. tenera* Tanita, 1940, and *L. ventosa* Hôzawa, 1940) were reported from the Japanese waters (Sagimi Sea, Wakayama Prefecture, Onagawa Bay, Mie Prefecture, Matsushima Bay, Izushima, Wagu Miye Prefecture, respectively). *Leucosolenia macquariensis* Dendy, 1918 was reported from the west coast of Macquarie Island; *L. australis* Brøndsted, 1931 was reported from Comau Fjord; *L. albatrossi* Hôzawa, 1918 was reported from Copper Island and the Komandorski Islands; *L. echinata* Kirk, 1893 and *L. rosea* Kirk, 1896 were reported from New Zealand; *L. lucasi* Dendy, 1891 was reported from Port Phillip Heads, Australia; *L. nautilia* Laubenfels, 1930 was reported from California, USA; and *L. feuerlandica* Tanita, 1942 was reported from Tierra del Fuego, South America. The *Leucosolenia* species reported from the coasts of Japan account for most species. The type specimens of new species were found in the Yellow Sea, very close to Japan.

## Materials and methods

The specimens were collected in a scallop-breeding pond from the Yellow Sea and were preserved in 75% ethanol. Two specimens were deposited in the Marine Biological Museum of the Institute of Oceanology in the Chinese Academy of Sciences (**IOCAS**), Qingdao, China.

For examination of the spicules, a small piece of specimen was cut and placed in a 1.5 mL microcentrifuge tube to which 1000 µL of sodium hypochlorite solution was added (Kersken et al. 2016). The mixture was then vortexed, placed at environmental temperature, and vortexed occasionally during incubation until it was completely lysed. Next, the sample was centrifuged at 8000 rpm for 2 min, the supernatant was poured off, 1000 µL of distilled water was added, and the sample was again centrifuged at 8000 rpm for 2 min. This procedure was repeated four times, then the spicules were washed three times with 96% ethanol and then the spicules were preserved in the third ethanol solution.

Scanning Electron Microscopy (SEM) was performed with a Hitachi S3400N. Preserved spicules for SEM were adhered to stubs with double-sided carbon conductive tape and coverslip. After dehydration, the spicules were coated with gold in a Hitachi MC1000 (LOPES 2018).

Measurements of at least 20 spicules of each type were performed using an optical microscope (Nikon Eclipse Ni) with a micrometric eyepiece. The length from the tip to the base and the thickness at the base of each actine were measured. The reported numbers refer to the range of measurements for each spicule type. Photographs were taken with a stereomicroscope (Zeiss Stemi 2000-c) and an optical microscope (Nikon Eclipse Ni-U) equipped with a digital camera to evaluate difference between the length of the unpaired and paired actines of each type of triactine. For comparison with the new species, we only selected those species of *Leucosolenia* reported from the Pacific Ocean.

## Results

### Systematics

#### Class Calcarea Bowerbank, 1862

**Subclass Calcaronea Bidder, 1898**

**Order Leucosolenida Hartman, 1958**

**Family Leucosoleniidae Minchin, 1900**

**Genus *Leucosolenia* Bowerbank, 1864**

##### Leucosolenia qingdaoensis sp. nov.

Taxon classificationAnimaliaLeucosolenidaLeucosoleniidae

2214C588-8741-5B07-8578-D62F623CBE67

http://zoobank.org/F0C1D83E-3940-4D4C-B0BB-60A379ED507D

**Figure 2.**
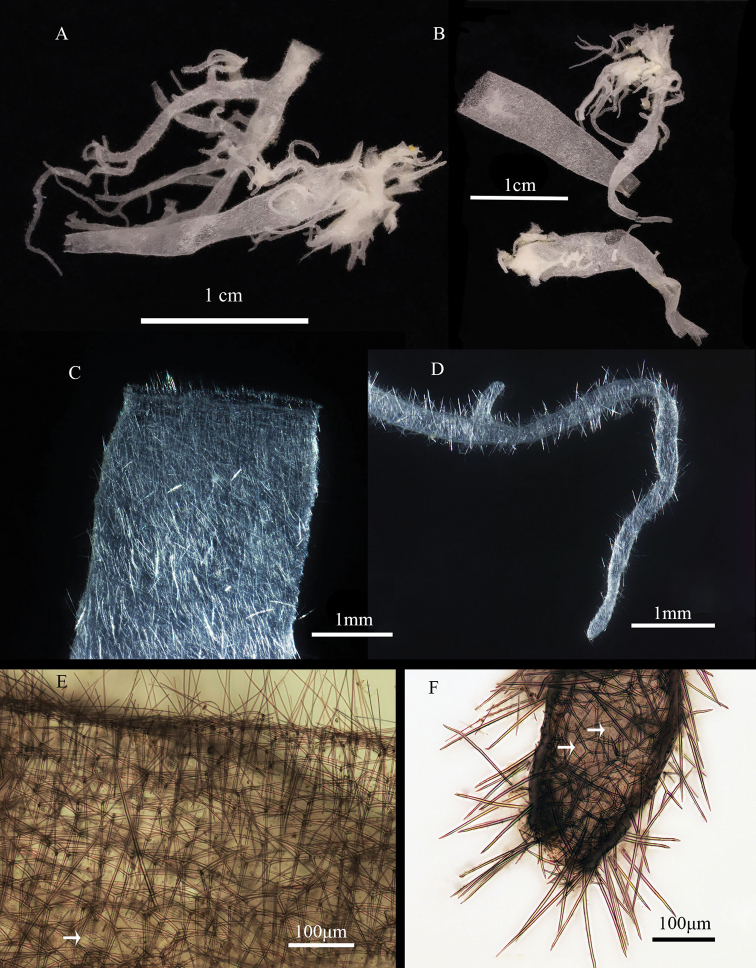
*Leucosolenia qingdaoensis* sp. nov. **A** holotype **B** paratype **C** detail of oscula (stereo microscope) **D** detail of root-like structures (stereo microscope) **E** detail of oscula (optical microscope) **F** detail of root-like structures (optical microscope); arrowhead pointing at the ostium.

**Figure 3.**
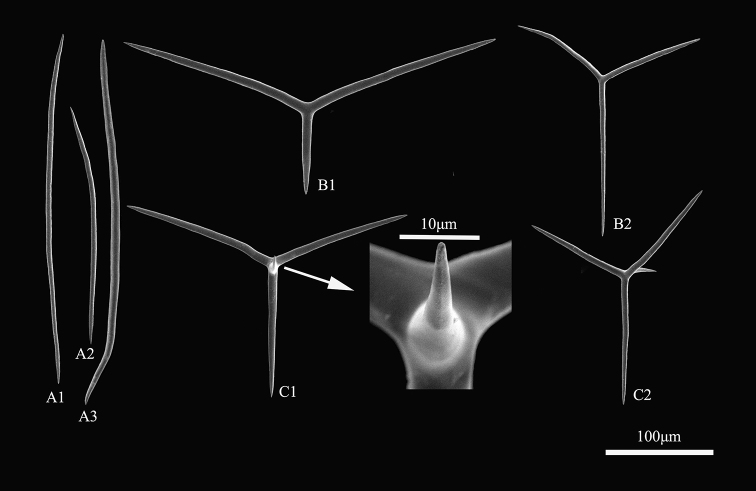
Spicules of *Leucosolenia qingdaoensis* sp. nov. (holotype) A1–3 = diactines; B1 = triactines of type 1; B2 = triactines of type 2; C1–2 = tetractines.

**Figure 4.**
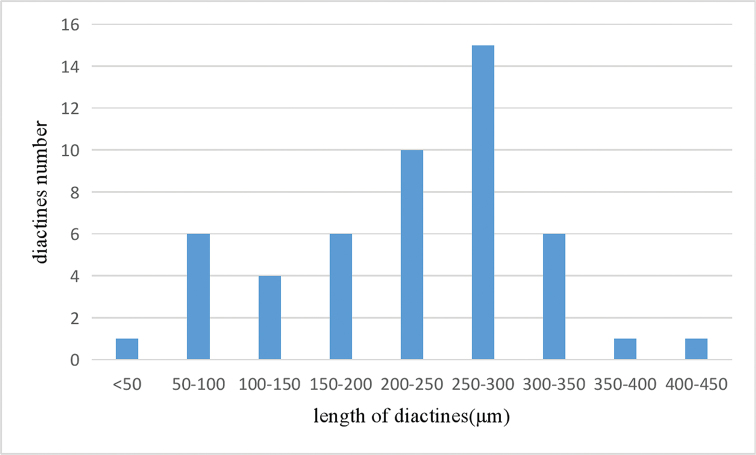
*Leucosolenia qingdaoensis* sp. nov. Size-class distribution of diactines (holotype).

Figs 1 , 2 , 3 , 4 ; Tables 1 , 2

###### Type material.

***Holotype***: MBM181606, scallop-breeding pond on southeastern Shandong Peninsula, China, June 1988, 0–0.3 m depth, collected by Shue Li, 35°58'N, 120°11'E. ***Paratype***: MBM181476, Zhonggang, Qingdao, China, 7 June 1984, 0–0.6 m depth, 36°06'N, 120°21'E.

###### Type locality.

Qingdao, Yellow Sea.

###### Etymology.

The name is derived from the type locality, Qingdao, China.

###### Description.

The sponge is arborescent, consisting of many thin-walled tubes, which are copiously ramified but never anastomosed. The sponge occurs as growth form. The oscula are terminal on erect tubes. The color of the sponge is white after being preserved in alcohol and in vivo. The external walls of the tubes are hairy, with diactines protruding at right or oblique angles from the body; the surface is minutely hispid, and the consistency is soft and fragile. The holotype measures 21.32 × 3.38 mm (height × width). The wall of the sponge body is very thin, and there is no fully developed inhalant system, the gap between the skeleton and the cell on the wall arrange evenly (Fig. 2F); only a small amount of cells is distributed on the thin sponge skeleton (Fig. 2C–F), which is a typical asconoid feature. All internal cavities of the sponge are lined by choanocytes.

###### Skeletal arrangement.

The skeleton consists of multifarious diactines, sagittal triactines of two types, sagittal tetractines with bent apical actines and triactine-like basal actines; together these form the wall of the ascon-type sponge body.

In the apical osculum (Fig. 2C, E), there are paired actines of triactines and tetractines, some additional tangential diactines, together forming a clear line dividing the apical oscula, and some radial diactines projecting beyond the apical osculum with different length.

In the sponge body (Fig. 2C, E), the triactines and tetractines are regularly arranged, their paired actines are parallel to the apical oscula, and the unpaired actines point downward, with slight folding allowed, but never overlapping; in contrast to the triactines and tetractines, the diactines are arranged more irregularly but generally point downward.

In the root-like structures (Fig. 2D, F), the arrangement of triactines and tetractines is the same as that in the body, but the arrangement of diactines is different; most of them tangentially project beyond the surface, which results in the surface having a slightly hispid appearance.

By observing the sponge tissue taken from different parts, it is clear that as the diameter of the tubes decreases, the contents of small diactines and small triactines increase. This observation can suggest that in the growth zone spiculogenesis is more intense.

###### Spicules.

***Diactines.*** There is only one type of diactine (Fig. 3A1–3), though the diactines vary in size and shape, their width varies from 24 µm to 61 µm, the length of diactines vary from 43 µm to 421 µm but half of the diactines present a length of 200–300 µm (Fig. 4). The shapes of the diactines are straight or slightly curved in different directions. The variation in *Leucosolenia* is very common and considerable.

***Triactines.*** Two types of triactines are present, with actines straight or undulated. Their ends are generally sharp or asymmetrical (Fig. 3B1–2). The paired actines are slightly curved. Some deformations are present.

Type 1: triactines with paired actines longer than unpaired actines (Fig. 3B1): unpaired actines 42–105 × 3–5 µm; paired actines 63–105 × 3–5 µm.

Type 2: triactines with unpaired actines longer than paired ones (Fig. 3B2): unpaired actines 76–129 × 3–4 µm; paired actines 60–104 × 3–4 µm.

***Tetractines.*** A relatively small number of tetractines are observed, approximately 10 per 100 spicules, with straight and fusiform actines (Fig. 3C1–2). The tetractines are similar to triactines but with the addition of apical actines, the apical actines are fairly stout and short, sharply pointed and curved: unpaired actines 93–119 × 2–5 µm; paired actines 50–93 × 2–5 µm; apical actines 11–29 × 2–5 µm.

###### Remarks.

Three species described by Tanita (*L. minuta*, *L. pyriformis*, and *L. serica*) exhibit only regular (equiangular and equiradiate) spicules. This characteristic does not fit the description of *Leucosolenia*, *L. qingdaoensis* sp. nov. can be easily differentiated from the 12 species of *Leucosolenia* reported from the Pacific Ocean. The skeletal compositions of these species are shown in Table 1.

**Table 1.** Spicules dimensions of *Leucosolenia* Bowerbank, 1864 in the Pacific Ocean. Measurements are reported in µm.

	**Triactines**		**Tetractines**			**Diactines**	**References**
	**Unpaired**	**Paired**	**Unpaired**	**Paired**	**Apical**		
	**Length/Width**	**Length/Width**	**Length/Width**	**Length/Width**	**Length/Width**	**Length/Width**	
*L. albatrossi*	70–90/8	80–100/8	70–90/8	80–100/8	40–60/6	70–90/8	Hôzawa 1918
	60–90/8	130–240/8	60–90/8	130–240/8	40–60/6–8	–	
*L. australis*	69–122/6	66–106/6	66–119/6	69–99/7	27–41/4	41–49/1	Azevedo et al. 2009
	–	–	–	–	–	63–347/7	
*L. echinata*	100/10	130/10	130/15	150/15	70/15	240–730/10–5	Kirk 1893
*L. eleanor*	80/7	80/7	140/9	140/9	140/9	105/4	Laubenfels 1932
	140/7	140/7	–	–	–	434/9	
*L. feuerlandica*	50–70/12–18	70–95/12–18	60–70/8–10	75–90/8–10	40–50/6–8	70–90/4–6	Tanita 1942
	60–70/8–10	75–90/8–10	–	–	–	–	
*L. lucasi*	100/5	70/5	100/5	70/5	<70/5	160/5	Dendy 1891
*L. macquariensis*	980/9	980/9	980/9	980/9	–	140/6	Dendy 1918
	–	–	–	–	–	90/5	
*L. minuta*	130–175/14–18	130–175/14–18	60–75/8–10	60–75/8–10	50–60/7–10	–	Tanita 1943
	60–75/8–10	60–75/8–10	–	–	–	–	
*L. mollis*	70–130/6–8	90–140/6–8	70–130/6–8	90–140/6–8	35–55/6	230–400/7–10	Tanita 1941
*L. nautilia*	140/9	140/9	140/9	140/9	30/8	400/10	Laubenfels 1932
	–	–	–	–	–	140/4	
	–	–	–	–	–	1000/20	
*L. pyriformis*	180–190/12–18	180–190/12–18	180–190/12–18	180–190/12–18	150–260/8–15	630–800/40–55	Tanita 1943
*L. rosea*	300/70	300/70	140/10	140/10	110/8	–	Kirk 1896
	200/18	200/18	–	–	–	–	
*L. serica*	140–210/7–8	140–210/7–8	140–210/7–8	140–210/7–8	90–135/8–10	–	Tanita 1942
*L. tenera*	80–180/7–10	90–210/7–10	80–180/7–10	90–210/7–10	30–10/6–8	200–530/8–12	Tanita 1940
*L. ventosa*	100–120/10	85–100/10	–	–	–	–	Hôzawa 1940
	150–180/20–25	140–150/20–25	–	–	–	–	
	100–120/10–14	70–90/10–14	–	–	–	–	
*L. qingdaoensis* sp. nov.	42–104/3–5	63–105/3–5	93–119/2–5	50–93/2–5	11–29/2–5	43–422/4–7	Present paper
	76–129/3–4	60–104/3–4	–	–	–	–	

The new species exhibits one type of diactine. In *L. ventosa* and *L. rosea*, there is no record of diactines, and in *L. mollis* and *L. nautilia*, there are two types of diactines. The triactines of *L. ventosa* are 2–8 times thicker than those in the new species; the triactines of *L. rosea* are 10–35 times thicker than in the new species; and *L. mollis* only has one type of triactine and all rays being nearly equally thick. The diactines of *L. nautilia* are extremely large, with a length of 1 mm and a thickness of 20 µm (Laubenfels 1932), while in the new species the diactines are less than 8 µm thick. Laubenfels (1932) gave few details on the actines, but *L. nautilia* differs from the new species by having only one type of triactine.

The difference between *L. albatrossi* and the new species is obvious. The diactines of *L. albatrossi* are club-shaped, while the diactines of the new species are spindle-shaped.

The sagittal triactines of the new species distinguish it from *L. macquariensis*, *L. tenera*, and *L. eleanor*. The new species have two types of sagittal triactines, while *L. macquariensis* and *L. tenera* only have one type of sagittal triactine, with rays of approximately equal length. *Leucosolenia eleanor* have both sagittal and regular triactines.

The new species, with slender and long diactines, the longest diactines 5 times longer than those of *L. feuerlandica*, is distinct from that species. Additionally, the triactines of the new species are sagittal, and the actines straight or undulated. However, the triactines of *L. feuerlandica* are pseudoderm sagittal and are tripod-shaped.

*Leucosolenia echinata*, *L. lucasi*, and *L. qingdaoensis* sp. nov. have many features in common, including their body shape, colour in alcohol, general arrangement, shape of diactines, and apical ray, but they show important differences in the shape of their triactines. The new species has two types of triactines; *L. lucasi* and *L. echinata* only have one type of triactine. The triactines of *L. lucasi* are sagittal, but the three angles are roughly equal; the triactines of *L. echinata* are generally regular, and frequently slightly sagittal, with the oral angle largest and the basal ray longest.

**Table 2.** Spicules measurements of *Leucosolenia qingdaoensis* sp. nov. (holotype).

	**length(µm)**				**width(µm)**				
	**min**	**mean**	**max**	**sd**	**min**	**mean**	**max**	**sd**	**n**
Diactines	43	219	422	*93*	1	4	7	*1.7*	50
Triactines 1									
paired	63	83	105	*9*	3	4	5	*0.8*	50
unpaired	42	66	105	*13*	–	–	–	–	–
Triactines 2									
paired	60	79	104	*11*	3	3	4	*0.4*	50
unpaired	76	102	129	*15*	–	–	–	–	–
Tetractines									
paired	50	77	93	*12*	2	4	5	*0.8*	20
unpaired	93	104	119	*11*	–	–	–	–	–
apical	11	21	29	*6*	–	–	–	–	–

#### Key to the species of *Leucosolenia* in the Pacific Ocean

**Table:** 

1	Skeleton contains only regular spicules	**2**
1a	Skeleton contains sagittal spicules	**4**
2	Skeleton including diactines	***L. pyriformis***
2a	Skeleton without diactines	**3**
3	Rays are stout	***L. minuta***
3a	Rays are relatively thin	***L. serica***
4	Skeleton contains diactines, triactines, and tetractines	**5**
4a	Skeleton contains triactines and tetractines	***L. rosea***
4b	Skeleton contains only triactines	***L. ventosa***
5	Skeleton contains one type of diactine	**8**
5a	Skeleton contains two types of diactines	**6**
6	Diactines are club-shaped	***L. macquariensis***
6a	Diactines are spindle-shaped	**7**
7	Skeleton without large diactines	***L. mollis***
7a	Skeleton including large diactines	***L. nautilia***
8	One tip of diactines has spines	***L. australis***
8a	Diactines have no spines	**9**
9	Skeleton contains one type of triactine	**10**
9a	Skeleton contains two types of triactines	**11**
10	Sagittal triactines with rays are of approximately equal in length	***L. tenera***
10a	Sagittal triactines with rays are of different lengths	***L. lucasi***
10b	Triactines are generally regular, slightly sagittal	***L. echinata***
11	Skeleton including tripod type of triactines	***L. feuerlandica***
11a	Skeleton without tripod type of triactines	**12**
12	Diactines have one ‘lance head’ type ends	***L. albatrossi***
12a	Diactines have two smooth and sharply pointed ends	**13**
13	Skeleton contains both sagittal and regular triactines	***L. eleanor***
13a	Skeleton contains only sagittal triactines	***L. qingdaoensis* sp.nov.**

## Supplementary Material

XML Treatment for Leucosolenia qingdaoensis
